# Lignocellulose-Degrading Microbial Communities in Landfill Sites Represent a Repository of Unexplored Biomass-Degrading Diversity

**DOI:** 10.1128/mSphere.00300-17

**Published:** 2017-08-02

**Authors:** Emma Ransom-Jones, Alan J. McCarthy, Sam Haldenby, James Doonan, James E. McDonald

**Affiliations:** a School of Biological Sciences, Bangor University, Bangor, Gwynedd, United Kingdom; b Microbiology Research Group, Institute of Integrative Biology, University of Liverpool, Liverpool, United Kingdom; c Centre for Genomics Research, Institute of Integrative Biology, University of Liverpool, Liverpool, United Kingdom; University of California, Berkeley

**Keywords:** *Bacteroidetes*, biomass, CAZymes, *Fibrobacter*, *Firmicutes*, landfill, cellulose degradation, cultivation, genomics, metagenomics, microbial ecology, *Spirochaetes*

## Abstract

The microbial conversion of lignocellulosic biomass for biofuel production represents a renewable alternative to fossil fuels. However, the discovery of new microbial enzymes with high activity is critical for improving biomass conversion processes. While attempts to identify superior lignocellulose-degrading enzymes have focused predominantly on the animal gut, biomass-degrading communities in landfill sites represent an unexplored resource of hydrolytic enzymes for biomass conversion. Here, to address the paucity of information on biomass-degrading microbial diversity beyond the gastrointestinal tract, cellulose (cotton) “baits” were incubated in landfill leachate microcosms to enrich the landfill cellulolytic microbial community for taxonomic and functional characterization. Metagenome and 16S rRNA gene amplicon sequencing demonstrated the dominance of *Firmicutes*, *Bacteroidetes*, *Spirochaetes*, and *Fibrobacteres* in the landfill cellulolytic community. Functional metagenome analysis revealed 8,371 carbohydrate active enzymes (CAZymes) belonging to 244 CAZyme families. In addition to observing biomass-degrading enzymes of anaerobic bacterial “cellulosome” systems of members of the *Firmicutes*, we report the first detection of the *Fibrobacter* cellulase system and the *Bacteroidetes* polysaccharide utilization locus (PUL) in landfill sites. These data provide evidence for the presence of multiple mechanisms of biomass degradation in the landfill microbiome and highlight the extraordinary functional diversity of landfill microorganisms as a rich source of biomass-degrading enzymes of potential biotechnological significance.

**IMPORTANCE** The microbial conversion of lignocellulosic biomass for biofuel production represents a renewable alternative to fossil fuels. However, the discovery of new microbial enzymes with high activity is critical for improving biomass conversion processes. While attempts to identify superior lignocellulose-degrading enzymes have focused predominantly on the animal gut, biomass-degrading communities in landfill sites represent an unexplored resource of hydrolytic enzymes for biomass conversion. Here, we identified *Firmicutes*, *Spirochaetes*, and *Fibrobacteres* as key phyla in the landfill cellulolytic community, detecting 8,371 carbohydrate active enzymes (CAZymes) that represent at least three of the recognized strategies for cellulose decomposition. These data highlight substantial hydrolytic enzyme diversity in landfill sites as a source of new enzymes for biomass conversion.

## Abstract (precis)

The microbial conversion of lignocellulosic biomass for biofuel production represents a renewable alternative to fossil fuels. However, the discovery of new microbial enzymes with high activity is critical for improving biomass conversion processes. While attempts to identify superior lignocellulose-degrading enzymes have focused predominantly on the animal gut, biomass-degrading communities in landfill sites represent an unexplored resource of hydrolytic enzymes for biomass conversion. Here, we identified *Firmicutes*, *Spirochaetes*, and *Fibrobacteres* as key phyla in the landfill cellulolytic community, detecting 8,371 carbohydrate active enzymes (CAZymes) that represent at least three of the recognized strategies for cellulose decomposition. These data highlight substantial hydrolytic enzyme diversity in landfill sites as a source of new enzymes for biomass conversion.

## INTRODUCTION

Biomass conversion and biofuel production from the microbial decomposition of lignocellulosic substrates are an attractive and sustainable alternative to fossil fuels (1). However, progress in this area has been hampered by the recalcitrance of lignocellulose, which requires expensive pretreatment (2, 3). Recent attempts to identify superior lignocellulose-degrading enzymes and microorganisms have focused predominantly on anaerobic gut environments such as the bovine rumen (4), elephant gut (5), and termite gut (6), in addition to biogas reactors (7). These environments harbor microbial communities that have evolved to attack lignocellulosic biomass without the pretreatments currently used in commercial processes (8).

Anaerobic plant biomass conversion is best studied in the rumen, where *Fibrobacter succinogenes*, *Ruminococcus albus*, and *Ruminococcus flavefaciens* are the predominant cellulolytic bacterial species (9). Recent advances in DNA sequencing technology, particularly for metagenome sequencing of microbial communities, have transformed our ability to characterize unexplored biomass-degrading diversity in anoxic environments (4, 6, 7). For example, Hess and colleagues (2011) performed deep metagenomic sequencing of the rumen cellulolytic community, generating 446 draft genomes (including 15 genomes from uncultivated species), and identified 27,755 putative carbohydrate active enzymes (CAZymes) (4), revealing significant potential of rumen microorganisms for biomass conversion. In addition, metagenome and metaproteome inventories of the termite hindgut also revealed a variety of cellulolytic bacteria and CAZYmes, many of which are related to those found in the rumen (6). More recently, metagenomic studies have utilized taxonomic binning to characterize the organisms involved in the anaerobic fermentation that occurs in biogas plants (10–12). However, there is still a paucity of information on the diversity and function of microorganisms in non-gut environments where cellulose hydrolysis occurs.

Landfill sites are highly heterogeneous, comprise mainly lignocellulosic material (13), and are therefore ideal environments for studying biomass conversion. In landfill, the decomposition of lignocellulosic biomass to methane is mediated by syntrophic interactions between hydrolytic (including cellulolytic) and fermentative bacteria, acetogenic bacteria, and methanogenic archaea (14). Members of the *Firmicutes* phylum associated with biomass conversion are consistently abundant in both culture-based (15, 16), and 16S rRNA gene (17–20) inventories of landfill sites, leading to the suggestion that they are the predominant degraders of biomass in landfill (15). However, members of the *Chlamydiae*/*Verrucomicrobia* group (17, 18), the *Cytophaga–Flexibacter–Bacteroides* group (17, 18), and phyla *Planctomycetes* (17), TM6 (19), *Chloroflexi* (19, 20), *Actinobacteria* (19), *Proteobacteria* (19, 20), *Lentisphaerae* (20), *Spirochaetes* (20), *Synergistetes* (20), *Thermotogae* (20), and *Fibrobacteres* (21) are also detected.

Cellulolytic clostridia (*Firmicutes* phylum), are more readily isolated from landfill sites and more amenable to PCR amplification, where they can represent as much as 100% of the sequencing output (13). However, it is clear that important cellulolytic functional groups in landfill sites have evaded detection by general 16S rRNA gene sequencing inventories. For example, novel *Fibrobacter* spp. (phylum *Fibrobacteres*, associated with cellulose hydrolysis in the rumen) were detected in landfill sites via the use of genus-specific 16S rRNA gene PCR primers (21), despite their absence from both 16S rRNA gene clone libraries (17, 18, 22) and studies utilizing 16S rRNA gene pyrosequencing (19, 20), with the exception of a single pyrosequencing study of landfill leachate (23). However, genus-specific quantitative PCR (qPCR) of landfill cDNA revealed that *Fibrobacter* spp. are abundant members of the landfill community, comprising ≤40% of the total 16S rRNA molecules in landfill (21). qPCR analysis of microbial DNA from heavily degraded cellulose (cotton) as studied here demonstrated that fibrobacters accounted for 29% of the total bacterial 16S rRNA gene copies, in comparison to members of the *Clostridia*, for which the highest relative abundance was that of *Clostridium* cluster III (17%) (24).

These taxa possess contrasting mechanisms for cellulose hydrolysis: *Firmicutes* utilize the cellulosomal method of cellulose decomposition (25), whereas *Fibrobacteres* possess fibro-slime proteins and pili for biomass attachment, followed by secretion of hydrolytic enzymes (26). Members of the *Bacteroidetes* are present in landfill sites (17–20), but their function is unknown; however, rumen *Bacteroidetes* possessing polysaccharide utilization loci (PUL) have recently been implicated in cellulose hydrolysis (27), suggesting a potential role in landfill cellulose decomposition. Whole-community metagenome sequencing studies can therefore obviate the biases associated with PCR and enable the simultaneous assessment of bacterial, eukaryotic, archaeal, and viral diversity, in addition to assigning function and generating taxonomic bins, informing follow-up attempts to isolate novel taxa (28, 29), and enabling the reconstruction of genomes (4). Hess et al. (4) utilized ultradeep metagenomic sequencing of a single pooled sample from the cow rumen in order to maximize the opportunity for genome reconstruction of individual members of that community, which resulted in the assembly of 15 genomes of uncultured microbial species. A similar approach has also been utilized to reconstruct genomes belonging to members of the *Fibrobacteres* phylum from metagenomic data derived from the termite gut, anaerobic digesters, and the ovine rumen (30).

Here, to address our hypothesis that landfill sites represent a repository of unexplored biomass-degrading diversity, we utilized a combination of 16S rRNA gene amplicon sequencing and shotgun metagenomics with taxonomic binning of reads to characterize the taxonomic and functional diversity of hydrolytic microbial communities on cotton (cellulose) baits in landfill leachate microcosms. The aims of our study were to (i) utilize 16S rRNA gene amplicon sequencing of replicated raw leachate and cellulose enrichment microcosm samples to identify members of the landfill microbiome that are significantly enriched with cellulose amendment and (ii) to identify the functional diversity and taxonomic identity of the landfill biomass-degrading microbiome using deep metagenomic sequencing and taxonomic binning. This study provides the first descriptions of functional diversity of landfill biomass-degrading communities, demonstrating the significant potential of landfill sites for the provision of novel CAZymes of ecological and biotechnological significance.

## RESULTS

### Bacterial community composition of raw landfill leachate and cellulose enrichment microcosms.

**FIG 1:**
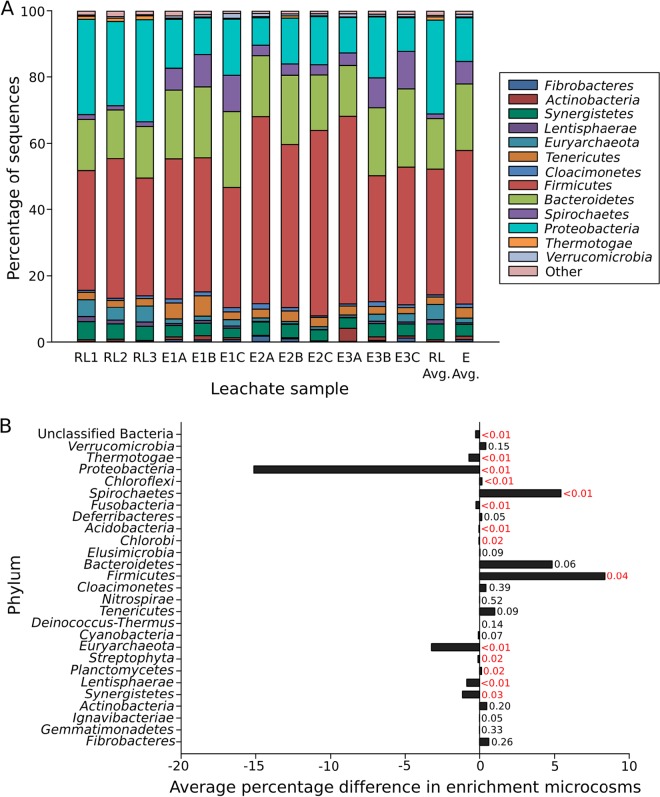
(A) Taxonomic identity at the phylum level of raw leachate (RL) and enrichment microcosms (E) as determined via Ion Torrent 16S rRNA gene amplicon sequencing and classification against the NCBI nucleotide database. The phyla shown are those that were >1% of the community. (B) Average percentage difference of phyla detected in Avicel enrichment microcosms in comparison to the raw leachate samples. Analysis of variance (ANOVA) was used to determine the significance between the samples, *P* values are shown on the bar plot; *P* values indicating a significant difference are shown in red.

The community composition of DNA extracted from three raw leachate samples and nine replicate landfill leachate microcosms containing 1% (wt/vol) Avicel was determined using 16S rRNA gene amplicon sequencing on the Ion Torrent PGM platform. On average, the raw leachate samples contained 26 phyla, whereas the Avicel enrichment microcosms contained an average of 23 phyla (Fig. 1). At the phylum level, the raw leachate and enrichment microcosm samples contained similar taxa; however, read counts for members of the *Firmicutes* (38.0 to 46.4%; *P* = 0.04), *Bacteroidetes* (15.2 to 20.0%; *P* = 0.06), *Fibrobacteres* (0.2 to 0.8%; *P* = 0.26), and *Spirochaetes* (1.4 to 6.8%; *P* = <0.01) increased in the Avicel enrichment microcosms, while members of the *Proteobacteria* (28.4 to 13.2%; *P* = <0.01) decreased (Fig. 1). At the family level, the raw leachate samples contained on average 90 families, in contrast to the Avicel-enriched microcosm samples, which contained an average of 68 families. Within the Avicel enrichment microcosms, members of the *Ruminococcaceae* (2.5 to 11.8%, *Firmicutes* phylum), *Clostridiaceae* (11.9 to 14.1%, *Firmicutes* phylum), *Bacillaceae* (1.8 to 6.2%, *Firmicutes* phylum), *Fibrobacteraceae* (0.1 to 0.7%, *Fibrobacteres* phylum), and *Spirochaetaceae* (1.3 to 6.5%, *Spirochaetes* phylum) were enriched in comparison to the raw leachate sample (see Table S1 in the supplemental material).

10.1128/mSphere.00300-17.2 TABLE S1  Family-level taxonomic classification of Ion Torrent 16S rRNA gene amplicons from raw leachate (RL) and Avicel enrichment (E) microcosms. Download TABLE S1, PDF file, 0.1 MB. Copyright © 2017 Ransom-Jones et al. 2017 Ransom-Jones et al. This content is distributed under the terms of the Creative Commons Attribution 4.0 International license.

### Microbiome analysis of colonized cotton biofilms from landfill leachate microcosms.

**FIG 2:**
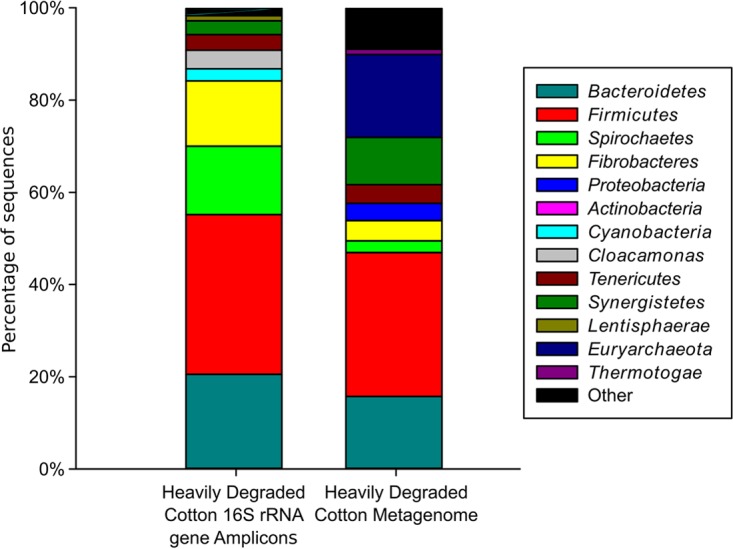
Phylum-level taxonomic distribution of the community of a heavily degraded cotton biofilm from landfill leachate microcosms as determined via 16S rRNA gene amplicon and metagenome sequencing. The phyla shown are those that were >1% of the community.

A dewaxed cotton string bait was incubated in a landfill leachate microcosm and retrieved for 16S rRNA gene amplicon and shotgun metagenome analysis after 6 weeks; visually, the cotton sample was heavily degraded and had little remaining structure when picked up with forceps. The colonized cotton biofilm, also analyzed by qPCR in a previous study (24), was subjected to DNA extraction and 454 pyrosequencing of 16S rRNA gene PCR amplicons (V1-to-V3 region) and shotgun metagenome sequencing. A total of 6,690 16S rRNA gene sequence reads were generated from the cotton biofilm and taxonomically assigned using the EzTaxon database. A rarefaction curve demonstrated that the majority of operational taxonomic units (OTUs) had been sampled (see Fig. S1 in the supplemental material), and the Shannon diversity index was 4.48. Nineteen phyla were detected in the 16S rRNA gene data set, with *Firmicutes* (37.4%), *Bacteroidetes* (20.9%), *Spirochaetes* (14.8%), and *Fibrobacteres* (14.2%) dominating the sequence reads (Fig. 2). These data are congruent with the major taxa associated with microbiome shifts in the replicated cellulose enrichment cultures described above. At the family level, *Ruminococcaceae* (24.1%, *Firmicutes* phylum), *Spirochaetaceae* (14.8%, *Spirochaetes* phylum), and *Fibrobacteraceae* (14.2%, *Fibrobacteres* phylum) (see Table S2 in the supplemental material) were the dominant taxa. The taxonomy of metagenome contigs assembled via Ray Meta (31) from the heavily degraded cotton sample was determined via comparison using One Codex (32) and classified against the One Codex database. A total of 63 phyla were identified in the metagenome data set, with the predominant phyla determined as *Firmicutes* (31.2%), *Euryarchaeota* (18.0%), *Bacteroidetes* (15.7%), *Synergistetes* (10.2%), and *Fibrobacteres* (4.4%) (Fig. 2).

10.1128/mSphere.00300-17.1 FIG S1  Rarefaction curve generated for the 454 16S rRNA gene amplicon data set generated from the heavily degraded cotton sample. Download FIG S1, PDF file, 0.1 MB. Copyright © 2017 Ransom-Jones et al. 2017 Ransom-Jones et al. This content is distributed under the terms of the Creative Commons Attribution 4.0 International license.

10.1128/mSphere.00300-17.3 TABLE S2  Family-level taxonomic classification of 16S rRNA gene pyrosequencing amplicons from the heavily degraded cotton biofilm sample. Download TABLE S2, PDF file, 0.05 MB. Copyright © 2017 Ransom-Jones et al. 2017 Ransom-Jones et al. This content is distributed under the terms of the Creative Commons Attribution 4.0 International license.

### Identification of CAZymes in the landfill microbiome.

**FIG 3:**
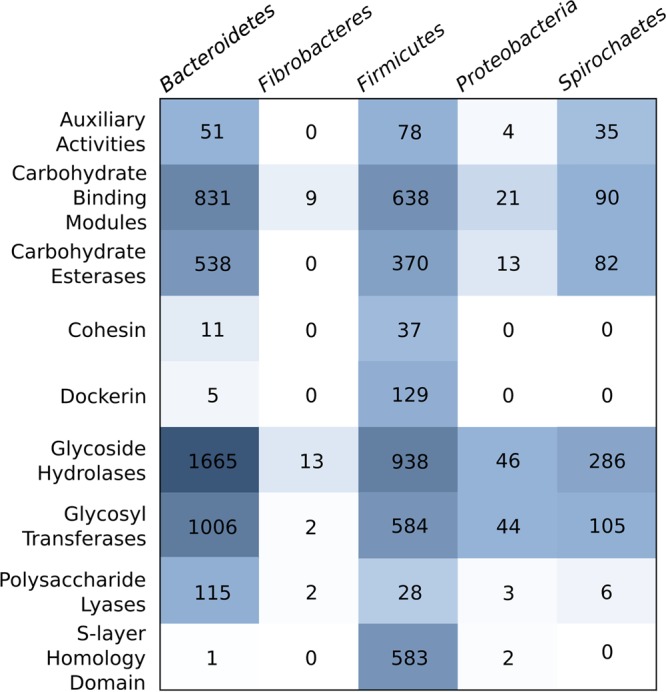
Number of carbohydrate active enzymes (CAZymes) detected in the *Bacteriodetes*, *Firmicutes*, *Spirochaetes*, *Proteobacteria*, and *Fibrobacteres* bins as determined via the dbCAN server with an E value of <1e–3.

CAZyme prediction on phylum-level binned metagenome contigs revealed 4,223 CAZymes in the *Bacteroidetes* bin, 3,385 in the *Firmicutes* bin, 604 in the *Spirochaetes* bin, 133 in the *Proteobacteria* bin, and 26 in the *Fibrobacteres* bin (Fig. 3). Hydrolytic enzyme systems are often modular in nature: for example, cellulosomes are multicomponent, multienzyme complexes found on the surface of cellulolytic bacteria and comprise a combination of catalytic enzymes, scaffold molecules, and carbohydrate binding molecules (carbohydrate binding modules [CBMs]) to maintain close contact with the substrate (25). The majority of CAZymes detected were glycoside hydrolases (GHs) (2,049 in the metagenome data set), glycosyl transferases (1,151), and CBMs (1,110), with auxiliary activity enzymes (113), carbohydrate esterases (634), cohesins (34), dockerins (85), polysaccharide lyases (107), and S-layer homology domains (320) also detected (Fig. 3). These enzymes included a number of GH families involved in lignocellulose degradation such as GH3, GH5, GH8, GH9, GH30, GH48, GH51, GH74, and GH94 (see Table S3 in the supplemental material), in addition to enzymes involved in the degradation of other polysaccharides (Table 1).

10.1128/mSphere.00300-17.4 TABLE S3  Detection of glycoside hydrolase (GH) families involved in lignocellulose hydrolysis in the *Bacteroidetes*, *Firmicutes*, *Spirochaetes*, *Proteobacteria*, and *Fibrobacteres* bins as determined via the dbCAN server. Download TABLE S3, PDF file, 0.1 MB. Copyright © 2017 Ransom-Jones et al. 2017 Ransom-Jones et al. This content is distributed under the terms of the Creative Commons Attribution 4.0 International license.

**TABLE 1:** Numbers of CAZymes and proteins associated with cellulose degradation identified in phylum-level bins constructed from the metagenome of a landfill leachate cellulose enrichment microcosm

CAZyme family	No. of CAZymes, proteins, or predicted genes				
	*Bacteroidetes*	*Fibrobacteres*	*Firmicutes*	*Proteobacteria*	*Spirochaetes*
Cellulases					
GH5	67	3	46	5	5
GH9	28	3	37		1
GH12	1	1			
GH30	13		6		1
GH44	5		2		
GH45		1			
GH48	1		3		
GH51	27		24		3
GH74	76		19		1
GH94	11		34		5
Hemicellulases					
GH2	108		36		17
GH8	15	2	6		
GH10	29		28		6
GH11	4		2		
GH26	30	2	21		1
GH31	30		26	1	10
GH39	4		8		
GH42	10		7	3	
GH43	184	2	42		13
GH53	16		5		
CBMs associated with cellulases					
CBM2			12		
CBM3	1		39		
CBM8	4				
CBM16	19		8	2	
CBM30	12	1	14		2
CBM37	7		2		8
CBM44	149		18		
CBM46	2		3		
CBM49			2		
CBM59	1				
CBM69	1				
Other cellulose-binding proteins					
Cohesin	11		37		
Dockerin	5		129		
SusC like (TonB-dependent receptor)	511			1	5
SusD like	334			1	

Total no. of predicted genes	62,632	204	82,534	4,945	19,832

### *Spirochaetes* CAZymes.

Six CAZyme families containing cellulolytic enzymes (GH5, GH9, GH30, GH51, GH74, and GH94), five families containing hemicellulases (GH2, GH10, GH26, GH31, and GH43), and two cellulose-binding CBM families (CBM30 and CBM37) were detected in the *Spirochaetes* phylum bin (Table 1). Annotation of this bin via PROKKA also identified 5 TonB-dependent receptor (SusC) coding domains (Table 1), which are predominantly involved in the polysaccharide utilization locus (PUL) exhibited by members of the *Bacteroidetes* (33), although they have also been identified in *Spirochaetes* (34).

### *Firmicutes* CAZymes.

Eight CAZyme families associated with cellulose degradation (GH5, GH9, GH30, GH44, GH48, GH51, GH74, and GH94) and 10 families associated with hemicellulase activity (GH2, GH8, GH10, GH11, GH26, GH31, GH39, GH42, GH43, and GH53) were detected in the *Firmicutes* phylum bin, as well as eight CBM families associated with binding cellulose (CBM2, CBM3, CBM16, CBM30, CBM37, CBM44, CBM46, and CBM49) (Table 1). In addition, 37 cohesin and 129 dockerin coding domains were also detected (Table 1), which are required for the assembly of cellulosomes that are utilized by the majority of cellulolytic clostridia for hydrolysis (35).

### *Fibrobacteres* CAZymes.

Four CAZyme families associated with cellulase activity (GH5, GH9, GH12, and GH45), three hemicellulases (GH8, GH26, and GH43), and a carbohydrate binding module 30 (CBM30) associated with cellulases were detected in the *Fibrobacteres* phylum bin (Table 1). Of particular interest is the detection of the GH45 cellulase exclusively in the landfill *Fibrobacteres* bin, as this CAZyme family has been found in all studied members of the *Fibrobacteres* and is thought to be distinctive to this group (30). In addition, 84 coding domains in the metagenome were identified as the *Fibrobacter succinogenes* major protein, a putative extracytoplasmic cellulose binding protein thought to be a cohesin analog (6, 30). All of these CAZyme families and associated proteins have been previously detected in the genomes of *Fibrobacteres* derived from cellulolytic environments of the rumen, termite gut, and anaerobic digesters and are now described for the first time in landfill site *Fibrobacteres*.

### *Bacteroidetes* CAZymes.

Nine CAZyme families associated with cellulase activity (GH5, GH9, GH12, GH30, GH44, GH48, GH51, GH74, and GH94) and 10 associated with hemicellulase activity (GH2, GH8, GH10, GH11, GH26, GH31, GH39, GH42, GH43, and GH53) were detected in the *Bacteroidetes* phylum bin (Table 1). Nine CBM families associated with cellulases (CBM3, CBM8, CBM16, CBM30, CBM37, CBM44, CBM46, CBM59, and CBM69) were detected, in addition to 11 cohesin and 5 dockerin coding domains (Table 1). Annotation of the metagenome via PROKKA also revealed the presence of a substantial number of coding domains corresponding to SusD family proteins (*n =* 334) and TonB-dependent receptors (SusC) (*n =* 511 [Table 1]), which are involved in the polysaccharide utilization locus (PUL) (33) and were recently hypothesized as being part of a novel mechanism for cellulose decomposition within the *Bacteroidetes* (27), alongside GH5, GH9, and GH94, which were also detected in this study. To our knowledge, this is the first evidence for cellulose degradation by *Bacteroidetes* in landfill sites.

## DISCUSSION

Previously, the composition of cellulolytic microbial communities in landfill sites had only been inferred through isolation studies (15), PCR inventories of specific taxa (21, 24, 34, 36), and 16S rRNA gene amplicon studies with “universal” primer sets (22). Here, we applied a “hook-bait” approach to enrich cellulolytic microorganisms from landfill leachate samples for taxonomic and functional analysis using 16S rRNA amplicon sequencing and shotgun metagenomics with taxonomic binning of reads. The use of a single metagenome sample to enable the reconstruction of genomes, as previously described by Hess et al. (4), resulted in a total of 371 individual genomes with low coverage due to the lack of available sequence data (see Table S4 in the supplemental material). However, this approach enabled binning of sequence reads from the phylum to species level and functional predictions of the role that key taxa play in cellulose hydrolysis in the landfill environment. This study therefore represents the first description of functional diversity in landfill biomass-degrading communities.

10.1128/mSphere.00300-17.5 TABLE S4  Identity and length (base pairs) of individual species-level bins generated from the metagenome data set. Download TABLE S4, PDF file, 0.1 MB. Copyright © 2017 Ransom-Jones et al. 2017 Ransom-Jones et al. This content is distributed under the terms of the Creative Commons Attribution 4.0 International license.

Here, a combined 16S rRNA gene amplicon and metagenome sequencing approach has demonstrated that *Firmicutes*, *Bacteroidetes*, *Spirochaetes*, and *Fibrobacteres* dominate the cellulolytic microbial community in landfill sites (Fig. 1). Raw leachate samples contained an average of 26 phyla, in comparison to the Avicel enrichment microcosms, which contained 23 phyla on average, with members of the *Firmicutes* (38.0 to 46.4%), *Bacteroidetes* (15.2 to 20.0%), *Fibrobacteres* (0.2 to 0.8%), and *Spirochaetes* (1.4 to 6.8%) enriched in the Avicel microcosms (Fig. 1). Members of the *Bacteroidetes* have previously been identified in landfill sites both via general bacterial 16S rRNA gene clone libraries (17, 18) and 454 pyrosequencing of 16S rRNA gene PCR amplicons (19, 20) and are known to occupy a variety of ecological niches, including activated sludge, decaying plant material, and compost (37). However, in addition to the decomposition of a range of polysaccharides, we provide the first detection of the major components of a *Bacteroidetes* cellulase system in landfill sites, suggesting a key role for *Bacteroidetes* in landfill cellulose decomposition.

Historically, *Firmicutes* have been considered the major degraders of cellulosic biomass in landfill sites (22), comprising 100% and 90% of 16S rRNA gene clones in libraries derived from solid cellulosic material and mixed cellulosic/leachate material, respectively, from a bioreactor treating landfill leachate (22). 454 pyrosequencing studies targeting the 16S rRNA gene have also detected both *Firmicutes* and, more specifically, *Clostridia* within an anaerobic bioreactor (19) and a lab-scale reactor treating landfill leachate (20), with *Clostridia* identified as the most abundant class within the *Firmicutes* (19, 20). Historically, anoxic environments are expected to contain large populations of clostridia, which are generally easier to isolate and cultivate than other obligate anaerobes of the *Bacteroidetes* and *Fibrobacteres*. Identification of members of the *Clostridia* as major components of landfill cellulolytic community supports previous qPCR analysis of this heavily degraded colonized cotton sample, where *Clostridium* clusters III, IV, and XIV totaled 21% of the 16S rRNA gene copies; however, it is significant that a greater proportion of 16S rRNA gene copies detected belonged to members of the genus *Fibrobacter* (29%) (24). Of the 19 recognized *Clostridium* clusters, four (I, III, IV, and XIVab) contain cellulolytic species (16), and it is likely that members of these clusters have played a role in the degradation of the cotton sampled here, with members of clusters III and IV most commonly identified in landfills (22, 24, 34, 38), in addition to cluster XIV (22, 24).

Metagenome analysis identified members of the *Firmicutes* (31.2%), *Euryarchaeota* (18.0%), *Bacteroidetes* (15.7%), *Synergistetes* (10.2%), and *Fibrobacteres* (4.4%) as the most abundant phyla in the cellulolytic biofilm (Fig. 2). While the presence of members of the *Firmicutes* and *Bacteroidetes* was largely consistent between the 16S rRNA gene amplicon and metagenome of the heavily degraded colonized cotton and the Ion Torrent sequence data, the distributions of other phyla differed between the two data sets (Fig. 2). Reads classified as *Spirochaetes* and *Fibrobacteres* were more prevalent in the 16S rRNA gene data set (14.8 and 14.2%, respectively) than the metagenome (2.6 and 4.4%, respectively) (Fig. 2) or Ion Torrent data (1.4 to 6.8% and 0.2 to 0.8% on average, respectively) (Fig. 1). The disparity between these results may be explained by the nature of the different sequencing approaches used and the underrepresentation of these phyla in genome databases. Despite the detection of *Fibrobacteres* in landfill sites via genus-specific 16S rRNA gene PCR primers (21, 24), they have remained undetected in this environment via either 16S rRNA gene clone libraries (17, 18, 22) or 454 pyrosequencing approaches (19, 20), resulting in a limited representation of the members of this phylum in sequence databases. Previous analysis of this data set against an earlier version of the One Codex database identified members of the *Fibrobacteres* as 0.1% of the total metagenome reads (data not shown). However, since this analysis was performed, Rahman et al. (30) utilized taxonomic binning to construct *Fibrobacter* genomes from metagenome data sets, resulting in the addition of seven new genomes to the One Codex database (*Fibrobacteria* bacterium genomes AD111, AD312, AD80, GUT221, GUT307, GUT31, and GUT77), and reanalysis of our metagenome data set increased the percentage of contigs assigned to the *Fibrobacteres* to 4.4% (Fig. 2), demonstrating that the lower relative abundance of poorly studied members of cellulolytic communities could potentially be due to underrepresentation in sequence databases, rather than these organisms playing a limited role in this environment. This phenomenon highlights the importance of further studies to enhance the representation of these taxa in the public databases.

Despite this, *Fibrobacteres* CAZymes associated with cellulase and hemicellulase activity, carbohydrate binding, and the *Fibrobacter succinogenes* major protein associated with cellulose binding were detected in the landfill *Fibrobacteres* phylum bin, demonstrating marked similarity to the repertoire of enzymes and proteins associated with cellulolytic members of the *Fibrobacteres* studied in the rumen, termite gut, and anaerobic digesters (30). These data add to the growing body of evidence that cellulose hydrolysis is a unifying feature of the *Fibrobacteres* phylum (30) and extend the ecological range of detection of the *Fibrobacter* cellulase system to include landfill sites. Given the absence of *Fibrobacter* spp. in other landfill 16S rRNA gene inventories (17–20, 22), due to their apparent underrepresentation by general bacterial primers, their detection here as the fourth most abundant phylum on highly degraded cotton is significant (14.2% of 16S rRNA gene sequences) (Fig. 2) and supports the assertion that fibrobacters are prevalent members of the landfill hydrolytic community (24). This is supported by previous qPCR analysis of the same cotton biofilm analyzed in this study, which determined that fibrobacters represented 29% of the total bacterial 16S rRNA gene copies (24). The abundance of cellulolytic fibrobacters in the landfill community is intriguing and potentially important, as Gullert et al. (7) reported a decreased richness of lignocellulolytic enzymes in biogas fermenters (compared with gut environments) due to low abundances of *Bacteriodetes* and *Fibrobacteres* and suggested that increasing the proportion of these taxa could potentially enhance hydrolytic performance. The abundance of landfill fibrobacters in this study therefore suggests that there is the potential to enhance future biomass conversion processes by using landfill-derived fibrobacters and *Bacteroidetes* as inocula.

*Spirochaetes* were the third most dominant phylum as determined via 16S rRNA gene amplicon sequencing (14.8%), and they were also abundant in the metagenome (2.6%) and Ion Torrent data set (average of 1.4 to 6.8%). Members of the *Spirochaetes* have been identified in 16S rRNA gene clone libraries (17, 18) and 16S rRNA gene pyrosequencing inventories (19, 20) of landfill sites, but their function is currently unknown. Spirochetes have also been isolated from the bovine rumen (39), and although those strains were not cellulolytic, they are capable of utilizing polymers such as xylan, pectin, starch, and cellobiose and may act in a symbiotic manner with cellulolytic organisms in order to improve the hydrolysis of cellulose (25). Kudo et al. (40) tested the cellulolytic capabilities of two rumen bacteria, *F. succinogenes* and *Ruminococcus albus*, both in pure culture and in coculture with *Treponema bryantii*, a spirochete. When grown in coculture with *T. bryantii*, both strains showed an increase in barley straw degradation and volatile fatty acid production compared to the pure cultures, despite the fact that *T. bryantii* is not capable of degrading cellulose, suggesting a symbiotic relationship between the organisms. In addition, transmission electron microscopy of the colonized barley straw showed that *T. bryantii* was closely associated with both *F. succinogenes* and the cellulose fibers (40). The importance of *Spirochaetes* in the rumen environment, their detection both here and in previous studies, and their close association with other members of the microbial community and the degraded cotton suggest that they are important symbiotic members of the anaerobic cellulose-degrading community in landfill sites.

Developments in taxonomic binning of metagenome data sets have transformed our ability to assign functional attributes of mixed microbial communities to specific taxa (41). Here, we utilized Taxator-tk to generate phylum-level taxonomic bins containing metagenome contigs for gene annotation and CAZyme profiling of each phylum. Phylum-level bins derived from the metagenome data set revealed that the *Bacteroidetes* bin contained the most CAZymes (4,223), compared to the *Firmicutes* (3,385), *Spirochaetes* (604), *Proteobacteria* (133), and *Fibrobacteres* (26) (Fig. 3), despite the fact that the *Firmicutes* were the dominant phylum within the metagenome (31.2% in comparison to 15.7% *Bacteroidetes*). While the detection of specific CAZyme groups and other elements of known cellulase systems in the phylum-level metagenome bins was largely congruent with the known composition of these systems described in other environments, it should be noted that for several reasons, including the potential lack of representation of landfill microbial genomes in the public databases, a proportion of sequences may have been incorrectly assigned to taxonomic bins. As discussed above, certain taxa are almost certainly underrepresented in the current publicly available databases—specifically those from landfill sites—and therefore it is likely that the CAZymes detected here represent only a fraction of the total present in these populations. This has been highlighted in this study, where the addition of eight additional *Fibrobacteres* genomes by Rahman et al. (30), increased the read-level composition of *Fibrobacteres* in the metagenome data set from 0.1 to 4.4%. This demonstrates the need for attempts to isolate and cultivate novel taxa from landfill sites and for the application of emerging technologies such as cell sorting and single-cell genomics, which could generate a step change in our knowledge of the landfill biomass-degrading community.

In total, 244 CAZyme families were identified in this study, including the families GH3, GH5, GH8, GH9, GH30, GH48, GH51, GH74, and GH94, which are associated with lignocellulose hydrolysis. The detection of these GH families is unsurprising given the high cellulosic content of landfill sites (13) and the fact that members of all of these GH families have been detected in similar studies on the bovine rumen (4), elephant gut (5), and a biogas reactor (7), and all but families GH30 and GH48 have also been identified in the hindgut of wood-feeding termites (6). In addition, cohesin and dockerin domains that mediate the assembly of cellulosomes in cellulolytic bacteria were also detected in the metagenome (Fig. 3), and the largest number of these genes was found in the *Firmicutes* bin; this is expected given that cohesins and dockerins are major components of cellulosomes that are exhibited by the majority of cellulolytic clostridia (42). Additionally, within the *Bacteroidetes* bin, both SusD family proteins and TonB-dependent receptors (SusC) were detected, which form part of the PUL that enables *Bacteroidetes* to degrade a variety of substrates (33). We have therefore demonstrated the presence of genes for at least three of the recognized microbial strategies for cellulose decomposition in the biosphere within landfill sites (the cellulosomal mechanism, the *Fibrobacteres* fibro-slime strategy, and *Bacteroidetes* PUL genes), highlighting the importance of landfill as an environment for the study of biomass decomposition.

### Conclusions.

Due to the recalcitrant nature of lignocellulosic substrates, understanding the diversity of microbial biomass conversion is a fundamental step toward unlocking their potential as a source for biofuel production. Recently, anaerobic environments such as the bovine rumen (4), elephant gut (5), termite gut (6), and a biogas reactor (7), where the microbial community has evolved to hydrolyze lignocellulose, have been identified as potential sources of novel enzymes. Here we established landfill sites as an unexplored and important source of novel hydrolytic diversity. We utilized a combination of molecular methods to characterize the cellulolytic biofilm of a heavily degraded cotton sample from a landfill leachate microcosm. These data demonstrated that members of the *Firmicutes*, *Bacteroidetes*, *Spirochaetes*, and *Fibrobacteres* are abundant in the landfill cellulolytic microbiome and possess an array of CAZymes that suggest an important role in the cellulose degradation that occurs in landfill sites. Additional Ion Torrent sequencing of 16S rRNA gene amplicons derived from raw leachate and Avicel enrichment micrososms also demonstrated enrichment of members of the *Firmicutes*, *Bacteroidetes*, *Fibrobacteres*, and *Spirochaetes* in the Avicel enrichment microcosms. This was further supported by metagenome sequencing of the heavily degraded cotton sample, which demonstrated the presence of members of the *Firmicutes* (31.2%), *Euryarchaeota* (18.0%), *Bacteroidetes* (15.7%), *Synergistetes* (10.2%), and *Fibrobacteres* (4.4%). Functional annotation of the total metagenome and phylum-level bins detected 244 CAZyme families, including members of families GH3, GH5, GH8, GH9, GH30, GH48, GH51, GH74, and GH94, which are known to be involved in cellulose degradation. Here, we report the first detection of the *Fibrobacter* cellulase system and the *Bacteroidetes* polysaccharide utilization locus (PUL) in landfill sites, providing evidence for the presence of multiple mechanisms of biomass degradation in the landfill microbiome. These data highlight landfill sites as a repository of unexplored biomass-degrading enzyme diversity, with potential application in the effective breakdown of recalcitrant lignocellulosic plant biomass for alternative fuel production and biotechnological processes.

## MATERIALS AND METHODS

### Construction of landfill leachate microcosms containing dewaxed cotton string.

The samples used in this study were obtained from microcosms previously described by McDonald et al. (24). Briefly, each microcosm was constructed in a sterile Nalgene carboy (10 liters) containing dewaxed cotton string (43) suspended in a nylon mesh bag. Microcosm 1 contained leachate from risers 3 and 4 of the Brombrough Dock landfill site (Wirral, United Kingdom), and microcosm 2 contained leachate from Brombrough Dock riser 5. The dewaxed cotton string was removed after 6 weeks of static incubation at ambient temperature and stored at −80°C prior to use as the source material for cultivation and DNA extraction.

### Sampling of landfill leachate and construction of microcosms containing Avicel.

Leachate samples were collected from the Hafod landfill site, Wrexham, United Kingdom. Three samples of landfill leachate were collected and transported to the laboratory, where they were stored at 4°C prior to analysis. A total of nine landfill leachate microcosms were established (three technical replicates for each of the three landfill leachate samples) in sterile 100-ml Nalgene bottles, each containing 1% (wt/vol) Avicel (Sigma). Each microcosm was inoculated with 100 ml of landfill leachate immediately after sampling and incubated for 2 weeks at 41°C.

### DNA extraction of leachate and microcosms containing Avicel.

Raw leachate samples were shaken gently, and 50 ml from each sample was removed and centrifuged at 5,000 rpm for 20 min. Subsequently, the supernatant was removed and DNA was extracted from the pellet using the PowerSoil DNA isolation kit (MoBio) according to the manufacturer’s instructions. After a 2-week incubation with cellulose enrichment, the microcosms were shaken to ensure the Avicel was suspended and 25 ml from each sample was removed (*n =* 9) and centrifuged at 5,000 rpm for 20 min prior to the removal of the supernatant. DNA was extracted from the cell/biomass pellets using the PowerSoil DNA isolation kit (MoBio) according to the manufacturer’s protocol.

### DNA extraction of colonized cotton from landfill leachate microcosms and bacterial cultures.

DNA was extracted from 1.5 ml of broth culture. Cell cultures were centrifuged at 14,000 rpm for 5 min, the supernatant was removed, and the cell pellet was resuspended in nuclease-free water (Bioline) to a final volume of 500 μl. DNA extraction from 0.5 g of colonized cotton was performed using the phenol-chloroform method of Griffiths et al. (44). DNA was visualized on a 1% agarose (Bioline) gel with HyperLadder 1kb (Bioline) as a marker. DNA concentrations were determined using the Qubit double-stranded DNA (dsDNA) BR assay kit (Life Technologies, Inc.) and a Qubit fluorometer (Life Technologies, Inc.).

### Metagenome sequencing and analysis of heavily degraded colonized cotton.

Total DNA extracted from the heavily degraded colonized cotton from microcosm 1 was utilized to generate three Nextera sequencing libraries with insert sizes of 300, 400, and 600 bp. DNA libraries were then sequenced on one lane of an Illumina HiSeq, generating paired-end libraries (2 × 100 bp), by the Centre for Genomic Research, Liverpool, United Kingdom. For the 300-, 400-, and 600-bp libraries, 135,007,994, 103,519,620 and 93,776,958 reads were obtained, respectively, representing a total of 84.6 Gbp of metagenome sequence data (see Table S5 in the supplemental material).

10.1128/mSphere.00300-17.6 TABLE S5  Assembly of heavily degraded colonized cotton metagenome sequences. Download TABLE S5, PDF file, 0.1 MB. Copyright © 2017 Ransom-Jones et al. 2017 Ransom-Jones et al. This content is distributed under the terms of the Creative Commons Attribution 4.0 International license.

Adapter sequences were removed using Cutadapt (version 1.2.1) (45) and trimmed via Sickle (version 1.2) (46) with a minimum window quality score of 20 and reads shorter than 10 bp removed. The three sequence libraries were combined and assembled via Ray Meta (31) (version 2.3.1, k-mer = 31) using the HPC Wales computing network. Raw reads and assembled contigs were uploaded as separate data sets to One Codex and classified against the One Codex database (32).

The assembled metagenome contigs were subjected to taxonomic assignment at the phylum level using the Taxator-tk (version 1.2.2) script binning-blast (41). Gene prediction was performed on both the whole-metagenome data set and phylum-level bins belonging to the *Bacteroidetes*, *Fibrobacteres*, *Firmicutes*, *Proteobacteria*, and *Spirochaetes* via Prodigal v2.6 (47) and annotated via the dbCAN server (48) for the presence of CAZymes with a cutoff E value of <1e−3. Additional annotation was performed using Prokka v1.11 (49).

### 454 pyrosequencing and analysis of general bacterial 16S rRNA gene PCR amplicons generated from a heavily degraded cotton biofilm.

DNA extracted from the heavily degraded cotton was subjected to PCR with barcoded general bacterial primers that targeted the V1-to-V3 region of the 16S rRNA gene designed by Chunlab, Inc. (Republic of Korea): forward primer B16S-F (5′→3′ sequence GAGTTTGATCMTGGCTCAG) and reverse primer B16 (5′→3′ sequence WTTACCGCGGCTGCTGG) by Chunlab, Inc., Republic of Korea. These PCR amplicons were then purified via the QIAquick PCR purification kit (Qiagen) before sequencing with the 454-GS FLX Titanium sequencing system by Chunlab, Inc.

The 16S rRNA gene sequences were processed to separate the samples via the barcodes before removal of the barcode, linker, and PCR primer sequences, quality filtering, and chimera detection and removal and clustered into OTUs at 97% sequence similarity via Chunlab, Inc. Sequences were classified via CLcommunity against the ExTaxon database using the default parameters. Rarefaction analysis was performed and the Shannon diversity index was calculated using the Ribosomal Database Project pipeline.

### Ion Torrent PGM sequencing of raw leachate and Avicel enrichment microcosms.

DNA from the three raw leachate samples and nine microcosms was subjected to PCR with 16S rRNA gene PCR primers 515F (5′-GTGCCAGCMGCCGCGGTAA-3′) and 806R (5′-GGACTACHVGGGTWTCTAAT-3′) using the HotStarTaq Plus master mix kit (Qiagen, USA). The PCR cycling conditions were 94°C for 3 min, followed by 30 cycles of 94°C for 30 s, 53°C for 40 s, and 72°C for 1 min, followed by a final elongation step at 72°C for 5 min. Sequencing was performed on an Ion Torrent PGM following the manufacturer’s procedure, and data were processed to remove barcodes, primers, sequences of <150 bp, sequences with ambiguous base calls and with homopolymer runs exceeding 6 bp, and chimeras by MR DNA (Shallowater, TX). Operational taxonomic units (OTUs) were defined by clustering at 97% similarity and taxonomically classified using BLAST search against the NCBI nucleotide database. Sequences with homology to chloroplast 16S rRNA genes, plant nuclear and mitochondrial 18S rRNA genes, and arthropod 18S rRNA genes were removed from the data set. Analysis of variance (ANOVA) was used to determine the effect of enrichment with Avicel on the phyla present in leachate microcosms.

### Accession number(s).

The sequence data from this study have been deposited under the NCBI BioProject no. PRJNA351238. Metagenome assembly and phylum-level taxonomic bins are available at https://github.com/emmarj/Metagenome.
