# Supplementary material for: Lignocellulose-Degrading Microbial Communities in Landfill Sites Represent a Repository of Unexplored Biomass-Degrading Diversity
Source: mSphere. 2017 Aug 2;2(4):e00300-17. doi: 10.1128/mSphere.00300-17 (PMC5541161; doi:10.1128/mSphere.00300-17)
Supplement: TABLE S1 [file sph004172335st1.pdf]

Supplementary Table 1.

	RL1	RL2	RL3	E1A	E1B	E1C	E2A	E2B	E2C	E3A	E3B	E3C
Desulfuromonadaceae	1.1	1.2	1.5	0.0	0.0	0.0	0.0	0.0	0.0	0.1	0.1	0.0
Fibrobacteraceae	0.2	0.2	0.1	0.7	0.5	0.7	1.7	0.9	0.2	0.2	0.5	1.1
Bacillidae	0.0	0.0	0.0	4.2	14.6	0.6	0.8	3.9	10.5	2.2	0.3	0.1
Spirochaetaceae	1.4	1.2	1.3	6.2	8.4	10.8	3.1	3.3	2.8	3.8	9.0	11.2
Cytophagaceae	1.4	1.8	1.7	6.7	4.1	4.8	0.5	0.7	1.8	1.9	4.6	4.9
Peptoniphilaceae	0.2	0.3	0.2	0.0	0.0	0.0	0.0	0.0	0.0	0.0	0.0	0.0
Peptococcaceae	1.8	1.7	1.6	1.7	2.3	2.7	2.0	1.7	2.3	2.6	3.4	4.3
Pseudomonadaceae	2.0	1.5	1.8	0.1	0.7	0.0	0.2	0.2	0.2	1.0	0.0	0.0
Aerococcaceae	0.0	0.1	0.1	0.0	0.0	0.0	0.0	0.0	0.0	0.0	0.0	0.0
Phycisphaeraceae	0.0	0.0	0.0	0.0	0.0	0.1	0.0	0.0	0.0	0.0	0.1	0.0
Prolixibacteraceae	0.3	0.5	0.5	0.2	0.2	0.2	0.1	0.1	0.1	0.1	0.2	0.2
Alteromonadaceae	0.6	0.6	0.5	0.2	0.0	0.1	0.3	0.3	0.0	0.0	0.2	3.7
Methanosaetaceae	0.1	0.2	0.1	0.0	0.1	0.1	0.0	0.0	0.0	0.0	0.1	0.2
Desulfobacteraceae	0.7	0.5	0.6	0.1	0.1	0.0	0.0	0.0	0.0	0.0	0.1	0.1
Methermicoccaceae	0.0	0.0	0.0	0.0	0.0	0.1	0.0	0.0	0.0	0.0	0.0	0.0
Chlorobiaceae	0.1	0.1	0.1	0.0	0.0	0.0	0.0	0.0	0.0	0.1	0.0	0.0
Rikenellaceae	6.0	5.0	5.6	8.3	8.6	11.1	9.5	9.3	8.5	8.6	9.9	11.1
Bacillaceae	1.7	2.0	1.7	2.2	3.4	1.8	23.4	5.4	9.6	3.2	3.0	4.3
Clostridiaceae	11.2	13.3	11.0	19.2	12.7	13.7	9.3	12.3	11.6	15.5	14.8	18.0
Moraxellaceae	1.6	1.3	1.5	0.0	0.1	0.1	0.1	0.2	0.0	0.1	0.0	0.1
Porphyromonadaceae	4.0	3.8	4.0	1.6	2.3	2.2	3.9	5.0	1.2	1.5	1.6	2.2
Chloroflexaceae	0.0	0.0	0.0	0.0	0.0	0.0	0.0	0.1	0.0	0.0	0.0	0.0
Micrococcaceae	0.2	0.2	0.2	0.6	0.6	0.5	0.1	0.0	0.1	0.3	0.8	0.6

Victivallaceae	0.9	0.6	0.8	0.0	0.0	0.1	0.1	0.1	0.1	0.1	0.1	0.0
Flavobacteriaceae	0.1	0.1	0.1	0.5	0.3	0.5	0.6	0.5	0.6	0.4	0.3	0.2
Deltaproteobacteria	0.1	0.0	0.0	0.0	0.0	0.0	0.0	0.0	0.0	0.0	0.0	0.0
Deferribacteraceae	0.1	0.1	0.1	0.4	0.2	0.2	0.3	0.2	0.3	0.2	0.2	0.2
Haloplasmataceae	0.2	0.3	0.2	0.2	0.1	0.0	0.2	0.1	0.1	0.1	0.1	0.0
Defluviitaleaceae	0.1	0.1	0.1	0.0	0.0	0.0	0.1	0.1	0.0	0.1	0.0	0.0
Rhodobacteraceae	0.1	0.2	0.1	0.6	0.7	0.3	0.2	0.8	0.2	1.7	0.4	0.0
Desulfovibrionaceae	0.5	0.5	0.4	0.0	0.1	0.1	0.0	0.1	0.0	0.1	0.1	0.1
Erysipelotrichaceae	0.7	1.0	0.8	0.5	0.6	0.5	0.6	0.5	0.1	0.4	0.8	0.5
Methanomicrobiaceae	0.5	0.3	0.2	0.3	0.2	0.3	0.3	0.2	0.3	0.2	0.3	0.5
Clostridiales	3.0	2.6	3.3	1.6	1.6	1.9	1.2	1.4	1.2	1.9	1.5	1.5
Piscirickettsiaceae	0.0	0.0	0.0	0.0	0.1	0.0	0.0	0.0	0.0	0.0	0.1	0.0
Characeae	0.1	0.1	0.1	0.0	0.0	0.0	0.0	0.0	0.0	0.0	0.0	0.0
Nitrospiraceae	0.0	0.0	0.0	0.0	0.1	0.0	0.0	0.0	0.0	0.0	0.0	0.0
Campylobacteraceae	6.9	6.5	8.9	4.3	0.8	10.0	0.3	2.2	1.2	1.5	7.8	0.2
Alcaligenaceae	0.9	1.1	0.8	1.4	2.3	1.1	1.2	3.0	2.2	1.6	2.6	0.5
Nitrosomonadaceae	0.3	0.2	0.4	0.2	0.1	0.1	0.0	0.1	0.0	0.0	0.4	0.2
Thermotogaceae	0.9	1.0	1.2	0.3	0.2	0.3	0.3	0.7	0.2	0.2	0.2	0.2
Enterobacteriaceae	0.4	0.5	0.5	1.4	0.6	1.3	1.8	2.3	1.2	1.3	1.1	1.1
Mycoplasmataceae	0.0	0.0	0.0	0.1	0.0	0.0	0.0	0.0	0.0	0.0	0.0	0.0
Bdellovibrionaceae	0.0	0.0	0.0	0.0	0.0	0.1	0.0	0.1	0.0	0.0	0.0	0.0
Unclassified bacteria	0.1	0.2	0.1	0.0	0.0	0.0	0.0	0.0	0.0	0.0	0.0	0.0
Hyphomonadaceae	0.0	0.0	0.0	0.0	0.0	0.0	0.0	0.0	0.0	0.0	0.2	0.0
Saprospiraceae	0.3	0.2	0.3	0.6	1.0	1.4	2.1	1.8	0.9	0.4	1.2	2.0
Peptostreptococcaceae	0.0	0.2	0.0	0.0	0.0	0.0	0.0	0.0	0.0	0.0	0.0	0.0
Sphingobacteriaceae	0.2	0.3	0.2	0.5	0.5	0.5	0.0	0.6	0.9	0.3	1.0	0.8
Hydrogenophilaceae	0.1	0.1	0.0	0.2	0.1	0.7	0.7	1.2	2.9	0.1	0.4	0.4
Brucellaceae	0.0	0.0	0.1	0.0	0.0	0.0	0.0	0.0	0.0	0.0	0.0	0.0
Intrasporangiaceae	0.0	0.1	0.0	0.0	0.0	0.0	0.0	0.0	0.0	0.0	0.0	0.0

Thioalkalispiraceae	0.0	0.0	0.0	0.0	0.0	0.0	0.0	0.1	0.2	0.0	0.0	0.0
Clostridiales family xiii. Incertae sedis	1.0	0.8	0.7	0.2	0.2	0.2	0.3	0.4	0.3	0.3	0.1	0.1
Cloacimonetes	0.6	0.6	0.8	1.2	1.0	1.3	1.7	1.0	0.4	0.7	1.4	1.0
Staphylococcaceae	0.0	0.0	0.0	0.0	0.0	0.0	0.0	0.0	0.0	0.0	0.0	0.1
Caulobacteraceae	0.0	0.1	0.1	0.1	0.0	0.2	0.0	0.1	0.0	0.0	0.0	0.0
Thermoanaerobacteraceae	0.9	1.0	1.1	0.7	1.0	1.0	0.5	0.2	0.3	0.3	1.3	0.7
Synergistaceae	5.4	4.6	4.2	3.3	3.2	2.9	4.0	3.9	3.0	3.1	4.0	3.6
Bacteroidaceae	1.7	1.6	1.9	0.8	0.6	1.1	1.0	1.3	0.5	0.6	0.7	1.1
Anaplasmataceae	0.0	0.0	0.0	0.0	0.3	0.0	0.2	0.0	0.2	0.3	0.1	0.0
Corynebacteriaceae	0.2	0.2	0.1	0.0	0.4	0.0	0.1	0.2	0.0	0.2	0.1	0.0
Oceanospirillaceae	2.5	2.1	3.2	0.3	0.0	0.2	0.5	0.1	0.5	0.0	0.6	0.0
Xanthomonadaceae	0.2	0.2	0.1	0.0	0.0	0.1	0.1	0.1	0.1	0.2	0.2	0.1
Gracilibacteraceae	0.2	0.2	0.1	0.1	0.1	0.3	0.5	0.6	0.2	0.2	0.1	0.2
Alcanivoracaceae	0.0	0.0	0.0	0.1	0.0	0.0	0.0	0.0	0.0	0.0	0.0	0.0
Planctomycetaceae	0.0	0.1	0.0	0.3	0.2	0.1	0.1	0.1	0.1	0.1	0.1	0.1
Idiomarinaceae	0.6	0.4	0.5	0.2	1.7	0.7	0.1	0.2	1.0	0.2	0.1	0.0
Chroococcales	0.1	0.1	0.1	0.0	0.0	0.0	0.0	0.0	0.0	0.0	0.0	0.0
Acidaminococcaceae	0.9	0.9	1.0	0.3	0.4	0.3	0.2	0.1	0.1	0.3	0.3	0.2
Opitutaceae	0.0	0.0	0.0	0.0	0.1	0.1	0.1	0.0	0.0	0.0	0.0	0.1
Dehalococcoidaceae	0.1	0.1	0.1	0.2	0.2	0.2	0.1	0.2	0.2	0.2	0.3	0.2
Oligosphaeraceae	0.7	0.4	0.5	0.5	0.7	0.5	0.1	0.0	0.0	0.1	0.6	0.6
Microbacteriaceae	0.0	0.1	0.0	0.0	0.0	0.1	0.1	0.1	0.0	3.3	0.2	0.0
Proteinivoraceae	0.0	0.0	0.0	0.0	0.0	0.0	0.0	0.0	0.0	0.2	0.0	0.0
Acetobacteraceae	0.6	0.6	0.7	0.0	0.0	0.0	0.0	0.0	0.0	0.0	0.0	0.0
Chlamydomonadaceae	0.1	0.1	0.1	0.0	0.0	0.0	0.0	0.0	0.0	0.0	0.0	0.0
Geobacteraceae	0.2	0.1	0.2	0.0	0.1	0.0	0.0	0.0	0.0	0.0	0.0	0.0
Myxococcaceae	0.0	0.0	0.0	0.0	0.0	0.2	0.0	0.0	0.0	0.0	0.0	0.0
Syntrophomonadaceae	3.1	3.2	3.0	0.5	0.5	0.3	0.3	0.5	0.3	0.4	0.3	0.5
Chitinophagaceae	0.0	0.0	0.0	0.0	0.0	0.0	0.0	0.2	0.1	0.1	0.0	0.0

Eubacteriaceae	2.1	3.5	2.5	2.2	2.2	4.3	5.0	5.6	4.5	10.5	2.7	2.9
Acidobacteriaceae	0.0	0.1	0.0	0.0	0.0	0.0	0.0	0.0	0.0	0.0	0.0	0.0
Vibrionaceae	0.0	0.1	0.1	0.0	0.0	0.0	0.0	0.0	0.0	0.0	0.0	0.0
Mariprofundaceae	0.0	0.1	0.0	0.0	0.0	0.0	0.0	0.0	0.0	0.0	0.0	0.0
Lachnospiraceae	1.2	1.4	1.1	0.1	0.1	0.1	0.1	0.2	0.1	0.0	0.0	0.1
Bacteriovoracaceae	0.0	0.0	0.0	0.2	0.0	0.0	0.0	0.0	0.0	0.1	0.0	0.0
Enterococcaceae	0.3	0.4	0.3	0.0	0.0	0.0	0.0	0.0	0.0	0.0	0.0	0.0
Gloeobacterales	0.1	0.1	0.0	0.0	0.1	0.0	0.0	0.0	0.0	0.0	0.1	0.1
Desulfomicrobiaceae	4.6	2.8	4.2	0.1	0.2	0.3	0.6	0.5	0.3	0.1	0.3	0.1
Prevotellaceae	0.1	0.1	0.1	0.0	0.0	0.0	0.0	0.0	0.0	0.0	0.0	0.0
Carnobacteriaceae	1.0	1.6	1.1	0.0	0.0	0.0	0.0	0.0	0.0	0.0	0.0	0.0
Clostridiales family xi. Incertae sedis	1.4	1.4	1.1	0.5	0.6	0.4	0.6	0.6	0.7	0.6	0.3	0.4
Thiotrichaceae	0.0	0.1	0.1	0.0	0.0	0.0	0.0	0.0	0.0	0.0	0.0	0.0
Methylobacteriaceae	0.0	0.0	0.0	0.0	0.0	0.0	0.0	0.0	0.0	0.1	0.0	0.0
Methanocorpusculaceae	3.6	2.7	3.7	0.4	0.4	0.5	0.3	0.3	0.2	0.1	0.8	0.8
Thermoplasmataceae	0.4	0.2	0.4	0.0	0.0	0.0	0.0	0.0	0.0	0.0	0.0	0.0
Ruminococcaceae	2.6	3.0	2.1	10.1	8.0	8.2	11.0	17.0	17.7	18.1	8.8	7.6
Bacillales	0.8	0.9	0.7	0.1	0.6	0.1	0.1	0.1	0.2	0.1	0.1	0.1
Methanobacteriaceae	0.5	0.5	0.5	0.6	0.4	0.9	0.4	0.1	0.3	0.3	0.9	0.9
Endomicrobia	0.0	0.0	0.0	0.0	0.1	0.0	0.0	0.0	0.0	0.0	0.1	0.1
Pinaceae	0.0	0.1	0.0	0.0	0.0	0.0	0.0	0.0	0.0	0.0	0.0	0.0
Cryomorphaceae	0.0	0.0	0.0	0.1	0.1	0.0	0.0	0.1	0.1	0.0	0.1	0.1
Pelobacteraceae	0.2	0.2	0.1	0.0	0.1	0.1	0.3	0.3	0.1	0.1	0.0	0.0
Marinilabiliaceae	1.2	1.3	1.1	0.5	0.7	0.9	0.6	0.7	0.3	1.0	0.7	0.9
Oscillatoriales	0.0	0.0	0.0	0.1	0.0	0.0	0.0	0.0	0.0	0.0	0.0	0.0
Paenibacillaceae	0.1	0.1	0.1	0.2	0.2	0.3	0.8	0.5	0.7	0.6	0.2	0.0
Gammaproteobacteria	0.0	0.0	0.0	0.0	0.0	0.0	0.0	0.0	0.0	0.3	0.0	0.0
Streptococcaceae	0.6	0.8	0.6	0.0	0.0	0.0	0.0	0.0	0.0	0.0	0.0	0.0
Leptospiraceae	0.1	0.1	0.1	0.1	0.0	0.1	0.0	0.0	0.0	0.0	0.0	0.1

Anaerolineaceae	0.0	0.0	0.0	0.0	0.0	0.0	0.0	0.1	0.0	0.1	0.0	0.1
Verrucomicrobiaceae	0.3	0.4	0.3	0.6	0.5	1.3	0.8	0.5	0.6	0.9	0.5	0.6
Alphaproteobacteria	0.0	0.1	0.0	0.0	0.0	0.0	0.0	0.0	0.0	0.3	0.0	0.0
Helicobacteraceae	0.1	0.1	0.1	0.5	0.6	0.6	0.5	0.6	0.4	0.2	0.8	1.1
Lactobacillaceae	0.1	0.1	0.1	0.0	0.0	0.0	0.0	0.0	0.0	0.0	0.0	0.0
Francisellaceae	0.0	0.0	0.0	0.0	0.0	0.0	0.0	0.0	0.0	0.0	0.1	0.0
Acholeplasmataceae	2.2	2.1	2.3	4.6	5.3	2.3	2.6	3.1	2.5	2.6	2.3	1.8
Leuconostocaceae	0.1	0.1	0.1	0.0	0.0	0.0	0.0	0.0	0.0	0.0	0.0	0.0
Sphingomonadaceae	0.0	0.0	0.0	0.0	0.0	0.0	0.0	0.0	0.0	0.1	0.0	0.0
Veillonellaceae	0.8	1.2	0.8	0.0	0.0	0.0	0.0	0.0	0.0	0.0	0.0	0.0
Desulfobulbaceae	3.0	2.7	2.9	0.0	0.0	0.0	0.0	0.1	0.0	0.1	0.1	0.0
Rickettsiaceae	0.2	0.3	0.3	0.0	0.0	0.0	0.0	0.0	0.0	0.0	0.0	0.0
Rhodospirillaceae	0.0	0.0	0.0	0.9	0.2	0.2	0.0	0.5	0.0	0.0	0.0	0.0
Erythrobacteraceae	0.0	0.0	0.0	0.0	0.0	0.0	0.0	0.0	0.0	0.1	0.0	0.0
Methanosarcinaceae	0.0	0.0	0.0	0.0	0.0	0.0	0.0	0.0	0.0	0.0	0.0	0.1
Comamonadaceae	1.0	0.9	0.9	0.2	0.3	0.1	0.8	0.2	1.3	0.2	1.7	0.0
Fusobacteriaceae	0.2	0.3	0.3	0.0	0.0	0.0	0.0	0.0	0.0	0.0	0.0	0.0
