# Supplementary figures and images for: Lignocellulose-Degrading Microbial Communities in Landfill Sites Represent a Repository of Unexplored Biomass-Degrading Diversity
Source: mSphere. 2017 Aug 2;2(4):e00300-17. doi: 10.1128/mSphere.00300-17 (PMC5541161; doi:10.1128/mSphere.00300-17)

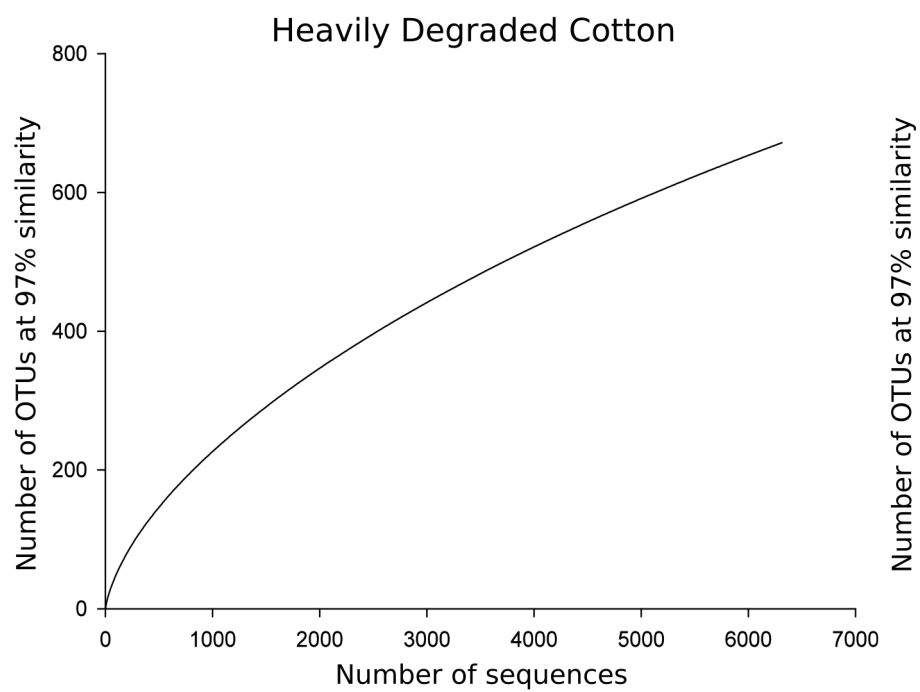

Supplement: FIG S1 [file sph004172335sf6.pdf]
