# Supplementary material for: Lignocellulose-Degrading Microbial Communities in Landfill Sites Represent a Repository of Unexplored Biomass-Degrading Diversity
Source: mSphere. 2017 Aug 2;2(4):e00300-17. doi: 10.1128/mSphere.00300-17 (PMC5541161; doi:10.1128/mSphere.00300-17)
Supplement: TABLE S2 [file sph004172335st2.pdf]

Supplementary Table 2.

Family	Heavily degraded cotton (% of classified reads)
Marinilabiliaceae	1.17
Oceanospirillaceae	0
Porphyromonadaceae	13.79
Flavobacteriaceae	3.18
Xanthomonadaceae	0.03
Microbacteriaceae	0.03
Comamonadaceae	0.09
Alcaligenaceae	0
Cryomorphaceae	0
Leptospiraceae	0.01
Cytophagaceae	0.28
Sphingobacteriaceae	0.79
Acholeplasmataceae	0.22
Chitinophagaceae	0.15
Rhodocyclaceae	0
Pseudomonadaceae	0.04
Rhodobacteraceae	2.35
Helicobacteraceae	0.04
Desulfovibrionaceae	0.09
Sphaerobacteraceae	0
Pseudoalteromonadaceae	0
Thiotrichaceae	0
Phycisphaeraceae	0
Saprospiraceae	0.09
Clostridiaceae	0.96
Ectothiorhodospiraceae	0
Campylobacteraceae	0.03
Deinococcaceae	0
Enterococcaceae	0
Leptotrichiaceae	0.09
Hahellaceae	0.03
Rikenellaceae	0.06
Puniceicoccaceae	0
Bacteroidaceae	0.21
Cyclobacteriaceae	0.04
Hyphomonadaceae	0
Cellulomonadaceae	0
Peptostreptococcaceae	0.1
Bacillaceae	0.45
Bifidobacteriaceae	0.01
Burkholderiaceae	0.01
Prevotellaceae	0.48
Piscirickettsiaceae	0
Sphingomonadaceae	0.03

Promicromonosporaceae	0
Alicyclobacillaceae	0
Prolixibacteraceae	0.33
Pseudonocardiaceae	0
Lachnospiraceae	2.98
Ruminococcaceae	24.15
Spirochaetaceae	14.81
Chromatiaceae	0.01
Halomonadaceae	0
Propionibacteriaceae	0
Phyllobacteriaceae	0
Oxalobacteraceae	0.03
Opitutaceae	0
Fibrobacteraceae	14.2
Xanthobacteraceae	0
Peptoniphilaceae	0.16
Neisseriaceae	0
Veillonellaceae	0.18
Lactobacillaceae	0.4
Rhodospirillaceae	0.03
Hyphomicrobiaceae	0
Eubacteriaceae	2.65
Planctomycetaceae	0.21
Methylococcaceae	0.03
Vibrionaceae	0
Streptococcaceae	0.07
Alteromonadaceae	0.06
Intrasporangiaceae	0.01
Methylobacteriaceae	0.01
Erysipelotrichaceae	0.81
Sanguibacteraceae	0
Thermoanaerobacteraceae	0.07
Paenibacillaceae	0
Chlorobiaceae	0
Flammeovirgaceae	0.1
Fusobacteriaceae	0
Desulfobacteraceae	0.01
Oscillospiraceae	0
Aurantimonadaceae	0
Halothiobacillaceae	0
Shewanellaceae	0
Idiomarinaceae	0
Anaerolineaceae	0.03
Lentisphaeraceae	0.15
Demequinaceae	0
Synergistaceae	2.86
Peptococcaceae	1.6
Acidithiobacillaceae	1.56
Planococcaceae	1.27

Mycoplasmataceae	0.24
Eggerthellaceae	0.12
Syntrophomonadaceae	0.09
Anaplasmataceae	0.07
Staphylococcaceae	0.07
Geobacteraceae	0.07
Francisellaceae	0.06
Acidaminococcaceae	0.06
Caldicoprobacteraceae	0.04
Fimbriimonadaceae	0.04
Myxococcaceae	0.03
Bartonellaceae	0.03
Spiroplasmataceae	0.03
Thermaceae	0.03
Thermodesulfobiaceae	0.03
Kosmotogaceae	0.03
Akkermansiaceae	0.03
Enterobacteriaceae	0.01
Methylocystaceae	0.01
Entomoplasmataceae	0.01
Bradyrhizobiaceae	0.01
Leuconostocaceae	0.01
Rhizobiaceae	0.01
Nocardioidaceae	0.01
Rhodobiaceae	0.01
Listeriaceae	0.01
Carnobacteriaceae	0.01
Acidobacteriaceae	0.01
Nautiliaceae	0.01
Symbiobacteriaceae	0.01
Sutterellaceae	0.01
Dehalococcoidaceae	0.01
Algiphilaceae	0.01
