# Supplementary material for: Lignocellulose-Degrading Microbial Communities in Landfill Sites Represent a Repository of Unexplored Biomass-Degrading Diversity
Source: mSphere. 2017 Aug 2;2(4):e00300-17. doi: 10.1128/mSphere.00300-17 (PMC5541161; doi:10.1128/mSphere.00300-17)
Supplement: TABLE S3 [file sph004172335st3.pdf]

Supplementary Table 3.

CAZy family	*Bacteroidetes*	*Fibrobacteres*	*Firmicutes*	*Proteobacteria*	*Spirochaetes*
AA2	8	0	0	3	0
AA3	3	0	8	0	2
AA4	0	0	8	0	1
AA6	38	0	61	1	32
AA7	2	0	1	0	0
CBM11	3	0	5	0	1
CBM12	0	0	2	0	0
CBM13	16	0	11	0	0
CBM14	0	0	1	0	0
CBM15	1	0	0	0	0
CBM16	19	0	8	2	0
CBM17	0	0	3	0	0
CBM2	0	0	12	0	0
CBM20	37	0	0	1	5
CBM21	0	0	1	0	0
CBM22	4	0	25	0	0
CBM23	3	0	5	0	2
CBM25	0	0	5	0	0
CBM26	10	0	2	0	0
CBM27	0	0	1	0	0
CBM28	0	0	3	0	0
CBM3	1	0	39	0	0
CBM30	12	1	14	0	2
CBM32	70	0	14	2	1
CBM34	0	0	14	0	3

CBM35	32	2	35	0	0
CBM36	0	0	2	0	0
CBM37	7	0	2	0	8
CBM38	0	0	0	0	0
CBM39	2	0	2	0	0
CBM4	39	1	19	3	2
CBM40	28	0	15	1	0
CBM41	1	0	1	0	1
CBM42	2	0	1	0	0
CBM44	149	0	18	0	0
CBM46	2	0	3	0	0
CBM48	59	1	22	3	5
CBM49	0	0	2	0	0
CBM5	0	0	1	0	0
CBM50	140	0	232	4	45
CBM51	0	0	2	0	0
CBM52	0	0	0	0	0
CBM53	1	0	1	0	0
CBM54	0	0	14	0	0
CBM56	10	0	8	1	2
CBM57	4	0	0	0	0
CBM59	1	0	0	0	0
CBM6	59	4	33	0	0
CBM61	13	0	2	0	0
CBM62	11	0	2	0	0
CBM63	0	0	3	0	0
CBM64	5	0	0	0	0
CBM65	0		10	0	0
CBM66	19		3	0	4

CBM67	32	14	0	1
CBM69	1	0	0	0
CBM70	0	0	0	0
CBM72	2	2	0	0
CBM74	0	0	0	0
CBM76	0	1	0	0
CBM77	8	1	0	0
CBM78	0	2	0	1
CBM79	0	5	0	0
CBM8	4	0	0	0
CBM9	24	15	4	7
CE1	167	74	4	23
CE10	117	52	0	21
CE11	19	0	1	0
CE12	30	24	0	4
CE13	0	1	0	0
CE14	10	14	0	1
CE15	13	2	0	0
CE16	1	0	0	0
CE2	11	18	0	0
CE3	34	38	2	7
CE4	41	99	5	5
CE6	49	14	1	1
CE7	23	16	0	3
CE8	22	2	0	4
CE9	1	16	0	13
Cohesin	11	37	0	0
Dockerin	5	129	0	0
GH1	0	11	0	8

GH10	29		28	0	6
GH100	1			0	0
GH103	0		0	1	0
GH105	45		13	0	8
GH106	18		0	1	0
GH107	0		0	0	0
GH108	4		0	1	0
GH109	76		83	7	35
GH11	4		2	0	0
GH110	2		0	1	0
GH112	0		3	0	0
GH113	0		7	0	0
GH114	2		0	0	0
GH115	20		4	0	0
GH116	0		2	0	0
GH117	9		1	0	0
GH119	1		0	1	0
GH12	1		0	0	0
GH120	0		11	0	0
GH121	1		0	0	0
GH122	1		0	0	0
GH123	2		1	0	0
GH124	0		6	0	0
GH125	2		2	0	0
GH126	0		2	0	0
GH127	28		12	0	3
GH128	7		0	0	0
GH129	0	0	2	0	0
GH13	90	0	73	4	37

GH130	41	0	25	0	4
GH133	22	0	4	1	2
GH15	17	0	2	0	0
GH16	40	0	7	0	4
GH18	7	0	47	0	2
GH19	3	0	1	2	0
GH2	108	0	36	0	17
GH20	24	0	2	1	9
GH23	61	0	30	4	10
GH24	2	0	3	2	0
GH25	3	0	14	0	0
GH26	30	2	21	0	1
GH27	11	0	2	0	0
GH28	58	0	6	3	4
GH29	22	0	12	0	0
GH3	59	0	59	1	23
GH30	13	0	6	0	1
GH31	30	0	26	1	10
GH32	8	0	8	0	0
GH33	20	0	8	1	1
GH35	8	0	3	1	0
GH36	6	0	8		5
GH37	1	0	0	0	7
GH38	5	0	3	1	1
GH39	4	0	8	0	0
GH4	0	0	15	0	12
GH42	10	0	7	3	0
GH43	184	2	42	0	13
GH44	5	0	2	0	0

GH45	0	1	0	0	0
GH48	1	0	3	0	0
GH49	3	0	0	0	0
GH5	67	3	46	5	5
GH50	2	0	0	3	0
GH51	27	0	24	0	3
GH52	0	0	0	0	0
GH53	16	0	5	0	0
GH55	1	0	0	0	0
GH57	22	0	2	0	16
GH63	4	0	2	0	0
GH65	13	0	14	0	4
GH66	2	0	1	0	0
GH67	9	0	3	0	1
GH73	20	0	14	0	6
GH74	76	0	19	0	1
GH75	0	0	0	0	0
GH76	12	0	4	0	0
GH77	15	0	18	1	17
GH78	42	0	17	0	1
GH79	1	0	0	0	0
GH8	15	2	6	0	0
GH81	1	0	1	0	0
GH82	0	0	1	0	0
GH88	11	0	4	0	3
GH89	1	0	0	0	0
GH9	28	3	37	0	1
GH91	0	0	1	0	0
GH92	34	0	1	0	0

GH93	2	0	3	0	0
GH94	11	0	34	0	5
GH95	28	0	8	0	0
GH97	42	0	0	0	0
GH98	2	0	0	0	0
GH99	12	0	0	0	0
GT1	2	0	12	0	1
GT10	2	0	0	0	1
GT11	2	0	2	0	0
GT12	1	0	1	0	0
GT13	7	0	0	0	0
GT14	0	0	1	0	0
GT17	0	0	1	0	0
GT19	22	0	16	1	0
GT2	316	2	164	13	11
GT20	11	0	0	0	0
GT21	0	0	1	0	0
GT22	2	0	0	0	0
GT23	2	0	1	0	0
GT24	1	0	0	0	0
GT25	3	0	0	0	0
GT26	4	0	19	0	6
GT27	19	0	1	0	0
GT28	45	0	56	2	17
GT3	24	0	0	0	0
GT30	20	0	1	1	0
GT31	1	0	1	0	0
GT32	2	0	2	0	0
GT33	5	0	0	1	0

GT35	25	0	29	0	9
GT39	0	0	4	0	0
GT4	306	0	175	20	35
GT40	0	0	0	0	1
GT41	6	0	0	0	1
GT44	0	0	2	0	0
GT45	3	0	2	0	1
GT46	0	0	1	0	0
GT5	40	0	27	2	12
GT51	42	0	28	1	5
GT52	0	0	1	0	0
GT56	4	0	0	0	0
GT6	2	0	0	0	0
GT60	0	0	1	0	0
GT61	1	0	0	0	0
GT62	0	0	2	0	0
GT64	1	0	0	0	0
GT66	2	0	0	0	0
GT7	7	0	0	0	0
GT70	1	0	2	0	0
GT71	1	0	0	0	0
GT73	1	0	0	0	0
GT74	2	0	0	0	0
GT76	0	0	1	0	0
GT78	0	0	0	0	0
GT8	4	0	3	0	2
GT81	0	0	2	0	0
GT83	31	0	12	1	2
GT84	0	0	10	0	0

GT87	0	0	1	0	0
GT9	26	0	1	2	0
GT90	2	0	0	0	1
GT92	2	0	0	0	0
GT94	6	0	0	0	0
GT95	0	0	1	0	0
PL1	33	2	2	0	0
PL10	5	0	5	0	0
PL11	23	0	2	0	2
PL12	7	0	8	1	2
PL14	3	0	1	0	0
PL15	0	0	0	1	0
PL17	1	0	2	0	0
PL18	0	0	1	0	0
PL21	1	0	0	1	0
PL22	19	0	1	0	0
PL24	1	0	0	0	0
PL3	1	0	0	0	0
PL5	2	0	0	0	0
PL6	2	0	0	0	0
PL7	2	0	0	0	0
PL8	1	0	0	0	0
PL9	14	0	6	0	2
SLH	1	0	583	2	0
