# Supplementary material for: Lignocellulose-Degrading Microbial Communities in Landfill Sites Represent a Repository of Unexplored Biomass-Degrading Diversity
Source: mSphere. 2017 Aug 2;2(4):e00300-17. doi: 10.1128/mSphere.00300-17 (PMC5541161; doi:10.1128/mSphere.00300-17)
Supplement: TABLE S4 [file sph004172335st4.pdf]

Supplementary Table 4.

Species	Total Length (bp)
Parabacteroides johnsonii	123
Conexibacter woesei	128
Leptotrichia buccalis	128
Pseudomonas aeruginosa	129
Streptococcus infantarius	129
Lactobacillus mucosae	130
Epulopiscium sp. 'N.t. morphotype B'	131
Ralstonia solanacearum	131
Candidatus Solibacter usitatus	132
Chitinophaga pinensis	132
Cellulophaga lytica	133
Methanococcus maripaludis	133
Thioalkalivibrio sulfidophilus	133
Paraprevotella xylaniphila	134
Arcobacter sp. L	135
Atopobium vaginae	136
Capnocytophaga sp. oral taxon 329	136
Geodermatophilus obscurus	136
Methylobacterium populi	136
Prevotella bryantii	136
Eubacterium rectale	137
Rhodobacter capsulatus	138
Streptococcus pneumoniae	138
Clostridium sp. L2-50	139
Clostridium sp. M62-1	140
Coxiella burnetii	140
Brachyspira murdochii	141
Prevotella copri	141
Streptococcus sanguinis	142
Clostridium carboxidivorans	144
Methanosarcina barkeri	144
Treponema azotonutricium	145
Roseburia inulinivorans	146
Leadbetterella byssophila	147
Mycobacterium marinum	147
Phyllobacterium sp. YR531	148
Thermoanaerobacter italicus	148
Bacillus azotoformans	149
Holdemania filiformis	149
Staphylococcus epidermidis	149
Thermoanaerobacterium thermosaccharolyticum	150
Anaeromyxobacter dehalogenans	152

Xylanimonas cellulosilytica	153
Selenomonas noxia	154
Caldisericum exile	155
Desulfobacterium autotrophicum	155
Legionella pneumophila	156
Joostella marina	157
Streptomyces venezuelae	157
Methanohalobium evestigatum	158
Anaerofustis stercorihominis	160
Lactococcus lactis	161
Selenomonas sp. CM52	162
Desulfonatronospira thiodismutans	164
Streptosporangium roseum	164
Gardnerella vaginalis	165
Clostridium acetobutylicum	167
Moorea producens	169
Bacteroides finegoldii	170
Psychromonas ingrahamii	170
Butyrivibrio proteoclasticus	171
Nitrolancetus hollandicus	174
Isoptericola variabilis	175
Prevotella melaninogenica	176
Lactobacillus acidipiscis	177
Caldicellulosiruptor saccharolyticus	181
Flavobacteriaceae bacterium HQM9	182
Methylomonas methanica	182
Chlorobium limicola	185
Rhodobacter sphaeroides	185
Oxalobacter formigenes	186
Methanosaeta concilii	188
Legionella drancourtii	189
Cecembia lonarensis	190
Desulfosporosinus youngiae	192
Bradyrhizobiaceae bacterium SG-6C	193
Kosmotoga olearia	193
Nocardia farcinica	193
Streptococcus mitis	193
Bartonella clarridgeiae	196
Turicibacter sanguinis	196
Anabaena sp. 90	197
Anaerobaculum hydrogeniformans	199
Lyngbya sp. PCC 8106	200
Desulfatibacillum alkenivorans	201
Magnetospirillum magneticum	201
Opitutaceae bacterium TAV1	201
Prevotella oris	202
Candidatus Zinderia insecticola	203

Bacteroides nordii	204
Clostridium symbiosum	209
Blautia hydrogenotrophica	210
Ilyobacter polytropus	213
Elusimicrobium minutum	216
Helicobacter pylori	216
Chthoniobacter flavus	217
Fusobacterium periodonticum	218
Buchnera aphidicola	219
Clostridium nexile	225
Leptospira noguchii	225
Fervidobacterium pennivorans	231
Leptospira weilii	234
Francisella novicida	236
Enterococcus faecium	237
Marivirga tractuosa	237
Methanocella conradii	247
Herpetosiphon aurantiacus	251
Geobacter sp. M21	252
Geobacillus sp. WCH70	257
Comamonas testosteroni	258
Lachnospiraceae bacterium 7 1 58FAA	258
Pontibacter sp. BAB1700	262
Methylocystis sp. SC2	264
Melioribacter roseus	265
Butyrivibrio crossotus	267
Methanothermococcus okinawensis	270
Paenibacillus mucilaginosus	275
Anaerococcus prevotii	276
Paenibacillus sp. JC66	277
Flavonifractor plautii	280
Pelodictyon phaeoclathratiforme	282
Rhodomicrobium vannielii	283
Indibacter alkaliphilus	287
Capnocytophaga canimorsus	289
unidentified eubacterium SCB49	289
Candidatus Sulcia muelleri	296
Desulfovibrio alaskensis	298
Methanococcoides burtonii	301
Capnocytophaga ochracea	302
Desulfosporosinus sp. OT	302
Syntrophothermus lipocalidus	302
Flavobacterium sp. CF136	303
Riemerella anatipestifer	303
Sebaldella termitidis	303
Desulfotomaculum ruminis	305
Caldithrix abyssi	308

Brachyspira intermedia	311
Roseburia hominis	311
Stigmatella aurantiaca	328
Lentibacillus sp. Grbi	331
Archaeoglobus fulgidus	332
delta proteobacterium MLMS-1	333
Gemmata obscuriglobus	337
Ruminococcus flavefaciens	344
planctomycete KSU-1	347
Anaerotruncus colihominis	353
Erysipelotrichaceae bacterium 5 2 54FAA	358
Clostridium sp. JC122	361
Borrelia burgdorferi	374
Ruminococcus lactaris	374
Bacteroides uniformis	375
Methanobacterium sp. AL-21	380
Thermacetogenium phaeum	380
Treponema denticola	381
Clostridium sp. DL-VIII	382
Faecalibacterium prausnitzii	382
Psychrobacter sp. 1501(2011)	385
Verrucomicrobiae bacterium DG1235	391
Bacteriovorax marinus	392
Tannerella sp. 6 1 58FAA CT1	393
Methanococcus voltae	394
Bacteroides ovatus	400
Hahella chejuensis	407
Clostridium sp. SY8519	417
Methylobacter tundripaludum	419
Phascolarctobacterium succinatutens	420
Bacteroides sp. 3 1 40A	421
Paenibacillus larvae	434
Clostridium kluyveri	435
Anaeromyxobacter sp. Fw109-5	436
Flavobacterium branchiophilum	438
delta proteobacterium NaphS2	442
Flavobacterium frigoris	443
Leeuwenhoekiella blandensis	445
Marvinbryantia formatexigens	446
Caldilinea aerophila	461
Brachyspira pilosicoli	462
Zunongwangia profunda	465
Haliscomenobacter hydrossis	482
Acaryochloris sp. CCMEE 5410	489
Clostridiales genomosp. BVAB3	489
Lactobacillus ruminis	491
Bacillus anthracis	496

Flavobacterium johnsoniae	496
Ktedonobacter racemifer	508
Thermosinus carboxydivorans	513
Clostridium beijerinckii	523
Clostridium spiroforme	535
Prevotella dentalis	544
Lachnospiraceae bacterium oral taxon 082	545
Wigglesworthia glossinidia	545
Methanolinea tarda	559
Aquimarina agarilytica	565
Methanolobus psychrophilus	569
[Bacteroides] pectinophilus	571
Cellulophaga algicola	583
Streptococcus infantis	589
Niastella koreensis	591
Fischerella sp. JSC-11	595
Methanosarcina mazei	603
Fusobacterium mortiferum	605
Clostridium papyrosolvens	616
Aequorivita sublithincola	622
Ignavibacterium album	629
Finegoldia magna	632
Halothermothrix orenii	640
Oribacterium sp. oral taxon 078	647
Campylobacter jejuni	661
Flavobacterium columnare	662
Spirochaeta smaragdinae	672
Escherichia coli	679
Ruminococcaceae bacterium D16	682
Candidatus Arthromitus sp. SFB-rat-Yit	684
Erysipelotrichaceae bacterium 6 1 45	689
Oscillatoria sp. PCC 6506	697
Rickettsia bellii	706
Methanococcus vannielii	713
Coraliomargarita akajimensis	727
Cyanothece sp. CCY0110	731
Tannerella forsythia	738
Lentisphaera araneosa	758
Dethiobacter alkaliphilus	768
Plesiocystis pacifica	774
Clostridium citroniae	791
Flavobacteriales bacterium ALC-1	793
Runella slithyformis	811
Fusobacterium nucleatum	818
Zymomonas mobilis	819
Peptostreptococcus anaerobius	847
Niabella soli	863

Bizionia argentinensis	879
Francisella tularensis	879
Verrucomicrobium spinosum	902
Clostridium asparagiforme	935
Desulfitobacterium dichloroeliminans	935
Clostridium butyricum	962
Sphingobacterium spiritivorum	990
Methanobrevibacter ruminantium	996
Clostridium acidurici	1,000
Treponema succinifaciens	1,017
Bacteroides cellulosilyticus	1,036
Myroides odoratimimus	1,046
Opitutaceae bacterium TAV5	1,048
Heliobacterium modesticaldum	1,051
Dyadobacter fermentans	1,053
Octadecabacter arcticus	1,073
Sphingobacterium sp. 21	1,077
Desulfosporosinus acidiphilus	1,087
Bacteroides caccae	1,110
Pedobacter heparinus	1,117
Methanosalsum zhilinae	1,143
Eubacterium hallii	1,149
Anaerobaculum mobile	1,168
Tepidanaerobacter acetatoxydans	1,178
Eubacterium saphenum	1,184
Pyramidobacter piscolens	1,290
Prevotella multisaccharivorax	1,321
Odoribacter splanchnicus	1,334
Methylobacterium nodulans	1,367
Cytophaga hutchinsonii	1,390
Clostridium leptum	1,433
Pedobacter agri	1,464
Geobacter metallireducens	1,506
Saccharophagus degradans	1,509
Opitutus terrae	1,524
Prochlorococcus marinus	1,548
Chlorobium chlorochromatii	1,589
Beggiatoa sp. PS	1,629
Desulfotomaculum gibsoniae	1,633
Bacteroides salanitronis	1,655
Paenibacillus lactis	1,700
Sphaerochaeta coccoides	1,725
Methanofollis liminatans	1,748
Pelosinus fermentans	1,750
Bacteroides fragilis	1,759
Desulfosporosinus orientis	1,771
Microscilla marina	1,838

Belliella baltica	1,929
Chlorobium phaeobacteroides	1,938
Candidatus Carsonella ruddii	1,969
Chloroherpeton thalassium	2,094
Clostridium clostridioforme	2,099
Emticicia oligotrophica	2,107
Clostridium cellulolyticum	2,129
Clostridium perfringens	2,130
Polaromonas sp. CF318	2,167
Brachyspira hyodysenteriae	2,199
Desulfotomaculum acetoxidans	2,208
Alkaliphilus oremlandii	2,261
Clostridium ljungdahlii	2,276
Methanosphaerula palustris	2,305
Pelobacter carbinolicus	2,307
Slackia heliotrinireducens	2,321
Bacteroides salyersiae	2,324
Caldicellulosiruptor bescii	2,329
Methanocorpusculum labreanum	2,342
Schlesneria paludicola	2,364
Dysgonomonas gadei	2,380
Lachnospiraceae bacterium 4 1 37FAA	2,533
Haloplasma contractile	2,560
Methanoregula boonei	2,718
Methanospirillum hungatei	2,750
Solitalea canadensis	3,007
Treponema primitia	3,072
Flavobacterium psychrophilum	3,160
Cellulosilyticum lentocellum	3,306
Bacteroides coprosuis	3,485
Flavobacteria bacterium BAL38	3,566
Selenomonas sp. oral taxon 137	3,633
Desulfitobacterium dehalogenans	3,652
[Clostridium] difficile	3,754
Oscillibacter valericigenes	3,804
Treponema brennaborense	3,815
Aminomonas paucivorans	4,056
Clostridium sp. BNL1100	4,074
Methanosarcina acetivorans	4,198
Fluviicola taffensis	4,450
Acinetobacter venetianus	4,543
Beggiatoa alba	4,743
Mahella australiensis	4,842
Dysgonomonas mossii	4,934
Pedosphaera parvula	4,994
Roseburia intestinalis	5,026
Anaerophaga sp. HS1	5,112

Acetobacterium woodii	5,452
Clostridium hathewayi	5,840
Parabacteroides goldsteinii	5,907
Mucilaginibacter paludis	5,960
Clostridium sp. D5	6,334
Eubacterium limosum	6,432
Bacteroides plebeius	6,844
Mesotoga prima	6,962
[Clostridium] sticklandii	7,042
Pseudoalteromonas spongiae	7,068
Galbibacter sp. ck-I2-15	7,071
Thermovirga lienii	7,614
Spirochaeta africana	7,649
Ruminococcus albus	7,672
Flexibacter litoralis	7,780
Enterococcus faecalis	8,513
Desulfitobacterium hafniense	9,078
Bacillus cereus	9,169
Clostridium phytofermentans	9,581
Clostridium clariflavum	9,857
Syntrophomonas wolfei	10,156
Syntrophobotulus glycolicus	10,534
Psychroflexus torquis	10,555
Cyclobacterium marinum	11,368
Clostridium saccharolyticum	13,098
Synergistes sp. 3 1 syn1	13,511
Staphylococcus aureus	13,944
Clostridium botulinum	14,346
Aminobacterium colombiense	15,814
Alkaliphilus metalliredigens	16,753
Marinilabilia salmonicolor	17,721
Lachnospiraceae bacterium 3 1 57FAA CT1	23,486
Methanoplanus limicola	25,338
Methanoplanus petrolearius	32,496
Paludibacter propionicigenes	33,520
Acetivibrio cellulolyticus	35,241
Sphaerochaeta pleomorpha	37,350
Acholeplasma laidlawii	41,838
Anaerophaga thermohalophila	48,672
Sphaerochaeta globus	64,187
Methanoculleus bourgensis	70,659
Clostridium thermocellum	118,201
Spirochaeta caldaria	151,879
Candidatus Cloacamonas acidaminovorans	160,661
Methanoculleus marisnigri	193,066
Dethiosulfovibrio peptidovorans	282,103
Fibrobacter succinogenes	287,828
