# Supplementary material for: Lignocellulose-Degrading Microbial Communities in Landfill Sites Represent a Repository of Unexplored Biomass-Degrading Diversity
Source: mSphere. 2017 Aug 2;2(4):e00300-17. doi: 10.1128/mSphere.00300-17 (PMC5541161; doi:10.1128/mSphere.00300-17)
Supplement: TABLE S5 [file sph004172335st5.pdf]

Supplementary Table 5.

Library	Initial number of reads	Number of reads after processing	Number of paired-end reads[a]	K-mer length used	Number of contigs	Average contig length (bp)	Largestcontig length (bp)
300 bp	135,007,994	134,585,268	67,087,235	61	115,162	780.4	134,352
400 bp	103,519,620	102,970,873	51,216,304	61	328,447	497.7	103,882
600 bp	93,776,958	92,853,901	45,970,929	61	102,639	826.5	91,326

a. Paired end reads consist of both a forward and reverse sequence, therefore each paired end read represents two of the sequences after processing.
